# Supplementary material for: Extrusion of subducted crust explains the emplacement of far-travelled ophiolites
Source: Nat Commun. 2021 Mar 8;12:1499. doi: 10.1038/s41467-021-21866-1 (PMC7940418; doi:10.1038/s41467-021-21866-1)
Supplement: Supplementary file 1 — Supplementary Information [file 41467_2021_21866_MOESM1_ESM.pdf]

Supplementary Information

Extrusion of subducted crust explains the emplacement of far-travelled ophiolites

Kristóf Porkoláb, Thibault Duretz, Philippe Yamato, Antoine Auzemery, Ernst Willingshofer

Supplementary Note 1: Explanation for the datasets collected from natural ophiolite belts (Fig. 1)

Datasets of 1) peak pressure-temperature (*P-T*) conditions, 2) duration of continental subduction-exhumation cycle, and 3) width of ophiolite sheets were collected from ophiolite belts worldwide in order to show key characteristics and provide a robust base for comparison with our numerical simulations.

Peak *P-T* conditions of the lower plate units in obduction systems were collected from 8 ophiolite belts, from multiple structural levels, when possible (Supplementary Table 1). The dataset shows the dominance of *HP-LT* metamorphism that is typical for subduction zones. Two outliers of high temperature-medium pressure (*HT-MP*) peak conditions have been reported from Cuba [1] and Eastern Anatolia [2]. These exceptions might be explained by oblique subduction below the oceanic upper plates and/or active rifting in the upper plate still ongoing during the initial stages of continental subduction [3,4].

The duration of continental subduction-exhumation cycle was assessed at each natural example based on a broad literature review focusing on 1) the timing of continental subduction initiation (following the subduction of the oceanic lower plate), that is in most cases constrained by the age of the youngest passive margin sediments accreted to the front of (or overridden by) the oceanic upper plate; 2) geochronological data that constrains the timing of prograde, peak, and retrograde metamorphism and/or cooling of the continental lower plate; 3) timing indications for the surface exposure of the previously subducted continental units (timing of erosion and/or deposition of unconformably overlying sediments). Both the initiation of continental subduction and the exhumation to surface/near surface conditions are loaded with timing uncertainties. For plotting, we used the best estimation of the time that passed from the initiation of continental subduction to surface/near surface exhumation of the continental lower plate units (colored circles on Fig. 1d). Error bars correspond to timing uncertainties that express the possible deviation from our best estimation allowed by available data. Sites where the uncertainty is well-constrained (enough data is available to bracket the uncertainty with confidence, and the interpretation of the data is not ambiguous), we used solid arrow lines for the plot (Fig. 1d). There are sites with very limited information available regarding the timing of exhumation (e.g. Hellenides, Brooks Range), or the interpretation of geochronological data that aims to constrain exhumation (retrograde metamorphism) is debatable (e.g. Southern Ural, Cuba, Brooks Range). In these cases, we used dashed arrow lines for plotting. For the data used for plotting and main references see Supplementary Table 2.

We further measured the width of far-travelled ophiolite sheets that are separated from their oceanic roots (open ocean or suture zone of former ocean) by the subducted and exhumed continental units (Fig. 1e). We measured the width in each cases along multiple sections (3-8 sections depending on the length of the ophiolite belt). The colored circle on the plot is the average of the measurements, while the error bars correspond to the maximum deviation from the average to show along-strike variation of the width. In cases where post-emplacement deformation disrupted the previously coherent far-travelled sheet, we took the sum of the smaller detached sheets along the sections. A special case of far-travelled ophiolite sheets is Anatolia, where unusually large amount of ophiolite is distributed over a 400 km wide area. Reconstructions imply, that all the ophiolites currently lying on top of the continent originated from a single ocean, and were later disrupted and distributed due to post-emplacement roof thrusting and extensional deformation [3,5]. Roof-thrusting of the ophiolites occurred during the subduction and exhumation of not only one, but three major continental units below the upper plate (Kırşehir/ Tavşanlı, Afyon, and Taurides thrust sheets) that subducted one-by-one, while the previously subducted continental unit(s) was already being exhumed as part of the upper plate [3]. We therefore took the sum of the disrupted smaller ophiolite sheets along several sections as the width of the far-travelled ophiolite sheet, which can reach 150 km, making the Anatolian sheet by far the largest (Fig. 1e). In case of New Caledonia, our far-travelled ophiolite sheet measurements took into account the offshore extrapolation of the sheet and the exhumed *HP-LT* units [6].

Supplementary Note 2: Convergence velocity test

Convergence velocity plays an important role in any geodynamic setting as it dictates the rate of tectonic movements and thus influences all the forces acting on the tectonic plates. Most notably, convergence velocity dictates strain rate, which has a major influence on stress distribution of the ductile lithospheric layers. Differential stress magnitudes in the ductile layers determine the degree of coupling between the brittle and ductile layers, thus strain rate (and so convergence velocity) is a key parameter in strain localization [7,8]. The continental crust in our model consists of an upper and a lower crust, both constituted by brittle and ductile layers (Fig. 2a). The coupling between these layers is substantially influenced by the strain rate (higher strain rate results in higher differential stress levels in the ductile layers, which results in higher degree of coupling). Our velocity test shows, that decreasing the convergence velocity leads to the extrusion of all the subducted continental upper crust (Supplementary Fig. 1a), while in our reference model some of the subducted upper crust remains underplated (Fig. 2e). This can be explained by the decreased coupling between brittle and ductile layers of the subducted crust due to the decrease in strain rate [8]. In contrast, increasing the convergence velocity results in increased strain rates and increased coupling in the subducted crust, and hence leads to delayed extrusion (Supplementary Fig. 1b), or no extrusion (Supplementary Fig. 1c) of the upper crust. It has to be noted, that higher convergence velocities (>= 5 cm/yr) could also produce extrusion of the upper crust similar to our reference model, if the crustal flow laws would be adjusted to the faster velocities (i.e. higher strain rates compensated by using weaker upper crustal material).

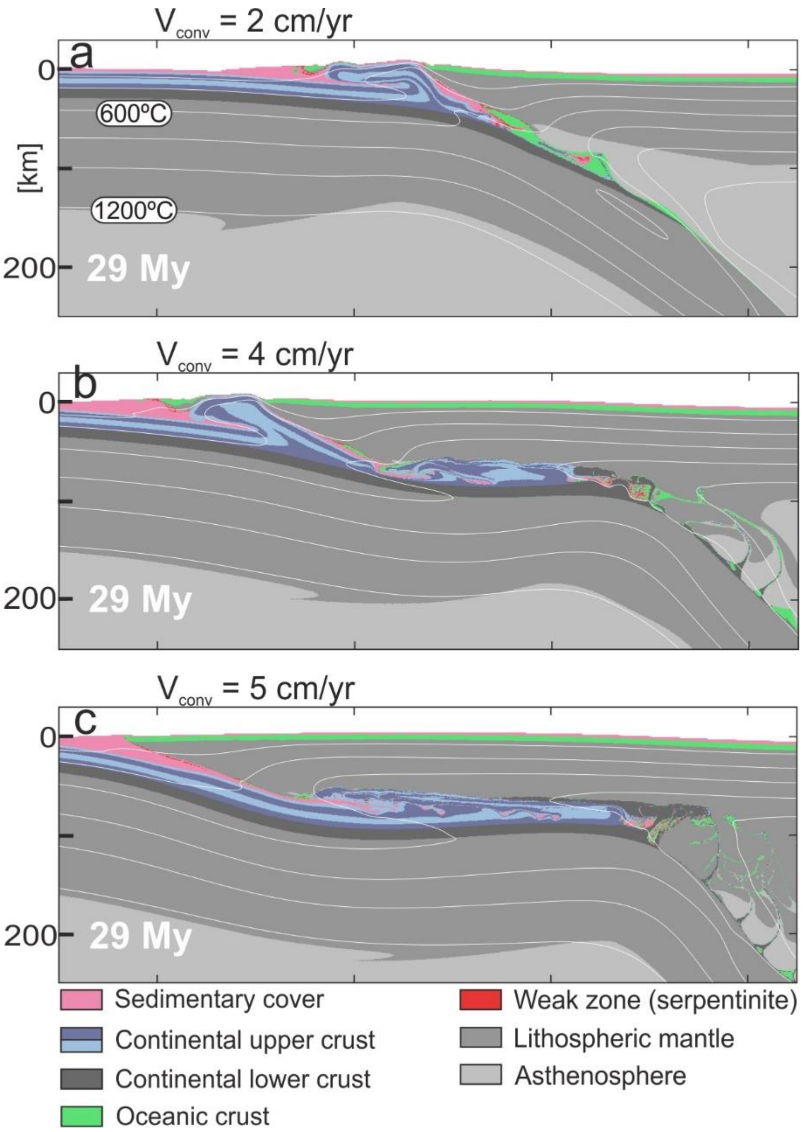

Supplementary Fig. 1 Convergence velocity test of the reference model (Convergence velocity of the reference model is 3cm/yr). a Model compositions at 29 Myr for the model variant with 2 cm/yr convergence velocity. **b** Model compositions at 29 Myr for the model variant with 4 cm/yr convergence velocity. **c** Model compositions at 29 Myr for the model variant with 5 cm/yr convergence velocity.

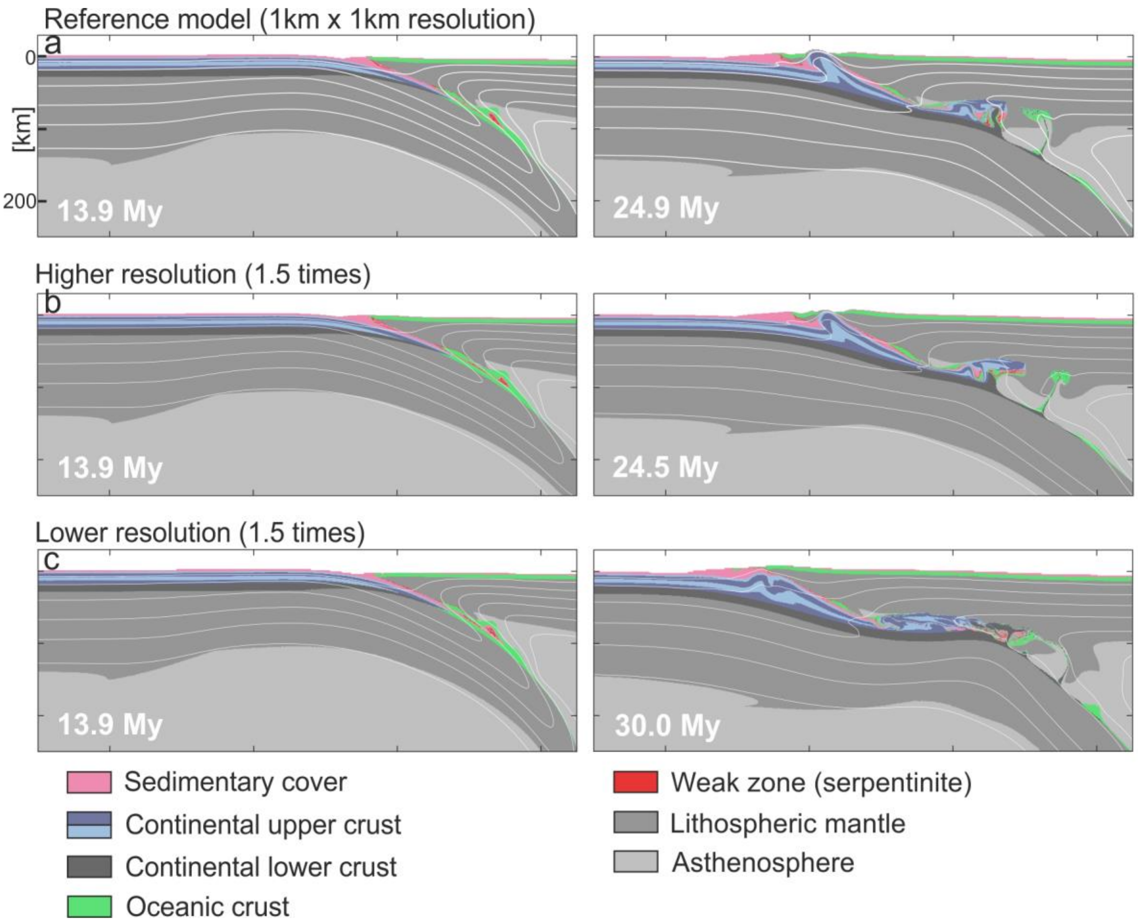

Supplementary Fig. 2 Resolution test of the reference model. a Reference model compositions at two snapshots. **b** Increased resolution model reproduces the reference model results with minimal differences confirming the accuracy of the results. **c** Decreased resolution model fails to reproduce reference model results as strain localization and nappe formation is prevented by the coarse resolution.

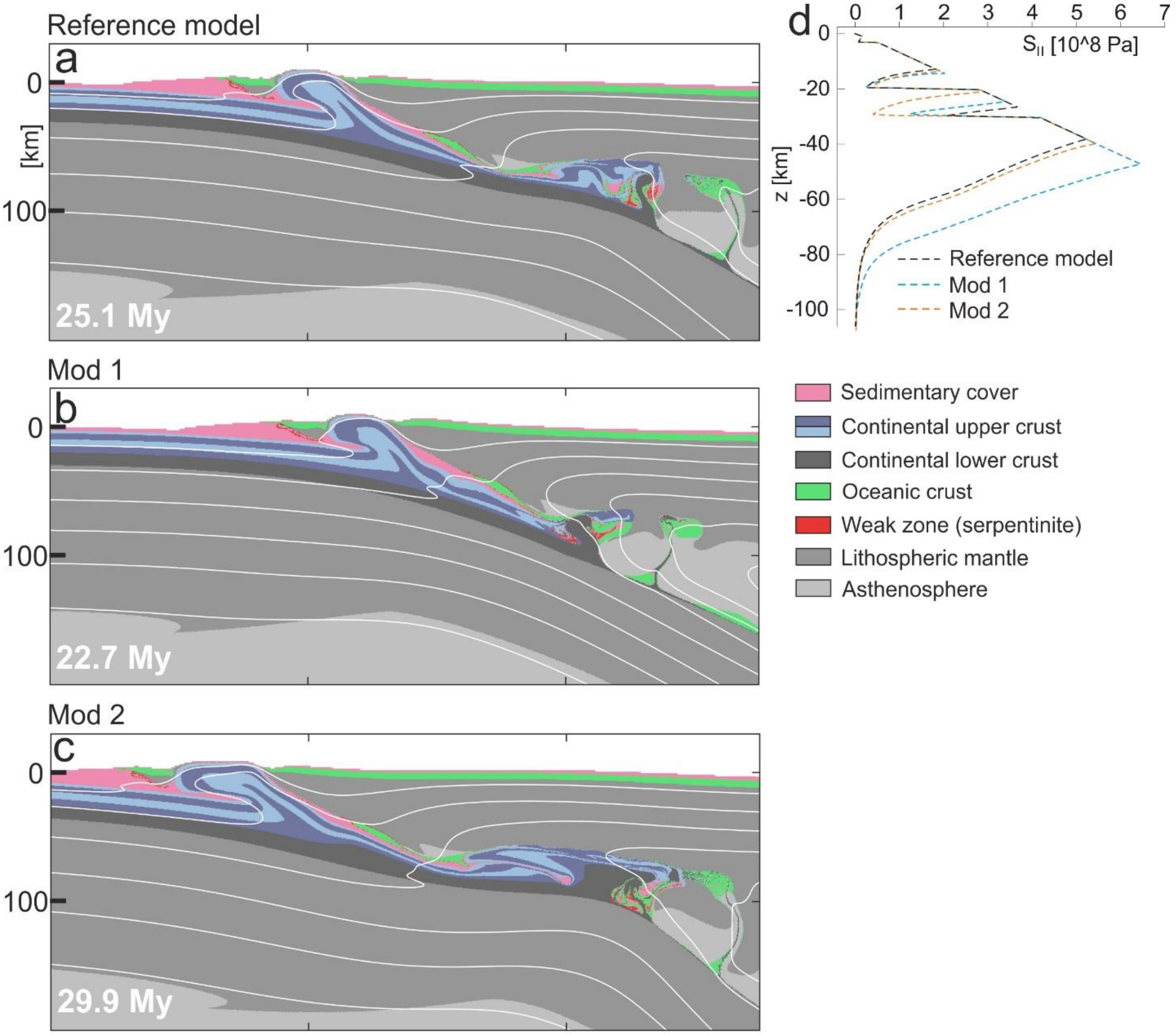

Supplementary Fig. 3 Timing and localization of upper crustal extrusion as the function of thermal and compositional changes in a decoupled continental lithosphere. a Reference model compositions at 25.1 My showing moderate amount of upper crustal underplating (~180 km long upper crust basement of the thinned passive margin), while the majority of the subducted upper crust is extruded upwards. For model parameters see Table 1. **b** Mod 1 variant of the reference model at 22.7 My where the heat production (Qr) of the continental crust was set to 0.5e-6 W.m^{-3}, and wet quartzite/felsic granulite flow laws [9] were used for the continental upper crust/lower crust. Almost the entire subducted upper crust is being extruded with minimal underplating. **c** Mod 2 variant of the reference model at 29.9 My where the heat production (Qr) of the continental crust was set to 1.5e-6 W.m^{-3}, and westerly granite/Maryland diabase flow laws [10,11] were used for the continental upper crust/lower crust. A significant portion of the subducted upper crust (~300 km) is not involved in extrusion and thus remains underplated below the oceanic upper plate. **d** Continental strength profiles (second stress invariant (Sii) vs depth (z)) of the three models (a, b, c), plotted for the initial conditions (t=0).

Location	T [°C]	P [GPa]	T_{error} [°C]	P_{error} [GPa]	Reference

Oman	525	2.3	25	0.1	[12]
Oman	300	1.05	5	0.05	[12]
New Caledonia	450	1.625	20	0.125	[13]
New Caledonia	535	2.4	15	0.15	[13]
New Caledonia	390	1.3	35	0.1	[14]
Cuba (Escambray)	570	2	40	0.3	[15]
Cuba (Pinos)	750	1.15	25	0.05	[1]
Kırşehir	700	0.8	20	0.05	[2]
Tavşanlı	430	2.4	30	0.3	[16]
Tavşanlı	275	1.2	25	0.1	[17]
Brooks range	475	1.1	25	0.2	[18]
Brooks range	400	1.05	30	0.15	[19]
Hellenides	500	2.35	50	0.15	[20]
Hellenides	460	1.22	30	0.1	[21]
Southern Ural	575	2.1	25	0.4	[22]

Supplementary Table 1. Pressure (P) and Temperature (T) data collected from the lower plate of natural ophiolite belts.

Location	t [My]	t + [My]	t - [My]	Reference
Brooks range	20	20	5	[23-25]
Carribian (Pinos and Escambray)	15	5	5	[26] and references therein
Kırşehir	17.5	10	5	[3] and references therein
Lesser Caucasus	12.5	5	5	[27-29]
New Caledonia	16	5	5	[6,30] and references therein
Oman	15	5	5	[12,31,32] and references therein
Hellenides-Dinarides	25	15	5	[21,33] and references therein
Quebec	10	5	2.5	[34-36]
Southern Ural	25	10	10	[37,38] and references therein
Tavşanlı	25	10	7.5	[3,39] and references therein

Supplementary Table 2. Best estimation of the continental subduction-exhumation cycle (t) based on available data, and timing uncertainties (t + and t -).

Supplementary References

1 García-Casco, A., Torres-Roldán, R., Millán, G., Monié, P. & Haissen, F. High-grade metamorphism and hydrous melting of metapelites in the Pinos terrane (W Cuba): Evidence for crustal thickening and extension in the northern Caribbean collisional belt. *Journal of Metamorphic Geology* **19**, 699-715 (2001).

2 Lefebvre, C., Peters, M. K., Wehrens, P. C., Brouwer, F. M. & van Roermund, H. L. Thermal history and extensional exhumation of a high-temperature crystalline complex (Hırkadağ Massif, Central Anatolia). *Lithos* **238**, 156-173 (2015).

3 van Hinsbergen, D. J. *et al.* Tectonic evolution and paleogeography of the Kırşehir Block and the Central Anatolian Ophiolites, Turkey. *Tectonics* **35**, 983-1014 (2016).
4 Plunder, A., Thieulot, C. & Van Hinsbergen, D. J. The effect of obliquity on temperature in subduction zones: insights from 3-D numerical modeling. *Solid Earth* **9**, 759-776 (2018).
5 Van Hinsbergen, D. J. *et al.* Orogenic architecture of the Mediterranean region and kinematic reconstruction of its tectonic evolution since the Triassic. *Gondwana Research* (2019).
6 Patriat, M. *et al.* New Caledonia obducted Peridotite Nappe: offshore extent and implications for obduction and postobduction processes. *Tectonics* **37**, 1077-1096 (2018).
7 Brun, J. P. Narrow rifts versus wide rifts: inferences for the mechanics of rifting from laboratory experiments. *Philosophical Transactions of the Royal Society of London. Series A: Mathematical, Physical and Engineering Sciences* **357**, 695-712 (1999).
8 Brun, J.-P. Deformation of the continental lithosphere: Insights from brittle-ductile models. *Geological Society, London, Special Publications* **200**, 355-370 (2002).
9 Ranalli, G. *Rheology of the Earth.* (Springer Science & Business Media, 1995).
10 Carter, N. L. & Tsenn, M. C. Flow properties of continental lithosphere. *Tectonophysics* **136**, 27-63 (1987).
11 Hansen, F. & Carter, N. in *The 24th US Symposium on Rock Mechanics (USRMS).* (American Rock Mechanics Association).
12 Agard, P., Searle, M. P., Alsop, G. I. & Dubacq, B. Crustal stacking and expulsion tectonics during continental subduction: P-T deformation constraints from Oman. *Tectonics* **29** (2010).
13 Brovarone, A. V. & Agard, P. True metamorphic isograds or tectonically sliced metamorphic sequence? New high-spatial resolution petrological data for the New Caledonia case study. *Contributions to Mineralogy and Petrology* **166**, 451-469 (2013).
14 Potel, S., Mählmann, R. F., Stern, W., Mullis, J. & Frey, M. Very low-grade metamorphic evolution of pelitic rocks under high-pressure/low-temperature conditions, NW New Caledonia (SW Pacific). *Journal of Petrology* **47**, 991-1015 (2006).
15 Stanek, K. P., Maresch, W., Grafe, F., Gravel, C. & Baumann, A. Structure, tectonics and metamorphic development of the Sancti Spiritus Dome (eastern Escambray massif, Central Cuba). *Geologica Acta* **4**, 151-170 (2006).
16 Okay, A. Jadeite–chloritoid–glaucophane–lawsonite blueschists in north-west Turkey: unusually high P/T ratios in continental crust. *Journal of Metamorphic Geology* **20**, 757-768 (2002).
17 Plunder, A., Agard, P., Chopin, C., Pourteau, A. & Okay, A. I. Accretion, underplating and exhumation along a subduction interface: from subduction initiation to continental subduction (Tavşanlı zone, W. Turkey). *Lithos* **226**, 233-254 (2015).
18 Gottschalk, R. R. & Oldow, J. S. Low-angle normal faults in the south-central Brooks Range fold and thrust belt, Alaska. *Geology* **16**, 395-399 (1988).
19 Patrick, B. High-pressure-low-temperature metamorphism of granitic orthogneiss in the Brooks Range, northern Alaska. *Journal of Metamorphic Geology* **13**, 111-124 (1995).
20 Mposkos, E. & Perraki, M. High pressure Alpine metamorphism of the Pelagonian allochthon in the Kastania area (Southern Vermion), Greece. *Bulletin of the Geological Society of Greece* **34**, 939-947 (2001).
21 Kilias, A. *et al.* Alpine architecture and kinematics of deformation of the northern Pelagonian nappe pile in the Hellenides. (2010).
22 Dobretsov, N. Blueschists and eclogites: a possible plate tectonic mechanism for their emplacement from the upper mantle. *Tectonophysics* **186**, 253-268 (1991).
23 Harris, R. Peri-collisional extension and the formation of Oman-type ophiolites in the Banda Arc and Brooks Range. *Geological Society, London, Special Publications* **60**, 301-325 (1992).
24 Wirth, K. R., Bird, J. M., Blythe, A. E., Harding, D. J. & Heizler, M. T. Age and evolution of western Brooks Range ophiolites, Alaska: Results from 40Ar/39Ar thermochronometry. *Tectonics* **12**, 410-432 (1993).

25 Hoiland, C. W., Miller, E. L. & Pease, V. Greenschist facies metamorphic zircon overgrowths as a constraint on exhumation of the brooks range metamorphic core, Alaska. *Tectonics* **37**, 3429-3455 (2018).
26 García-Casco, A., Iturralde-Vinent, M. A. & Pindell, J. Latest Cretaceous collision/accretion between the Caribbean Plate and Caribeana: origin of metamorphic terranes in the Greater Antilles. *International Geology Review* **50**, 781-809 (2008).
27 Hässig, M. *et al.* New structural and petrological data on the Amasia ophiolites (NW Sevan–Akera suture zone, Lesser Caucasus): insights for a large-scale obduction in Armenia and NE Turkey. *Tectonophysics* **588**, 135-153 (2013).
28 Hässig, M., Rolland, Y., Duretz, T. & Sosson, M. Obduction triggered by regional heating during plate reorganization. *Terra Nova* **28**, 76-82 (2016).
29 Rolland, Y., Galoyan, G., Sosson, M., Melkonyan, R. & Avagyan, A. The Armenian Ophiolite: insights for Jurassic back-arc formation, Lower Cretaceous hot spot magmatism and Upper Cretaceous obduction over the South Armenian Block. *Geological Society, London, Special Publications* **340**, 353-382 (2010).
30 Lagabrielle, Y., Chauvet, A., Ulrich, M. & Guillot, S. Passive obduction and gravity-driven emplacement of large ophiolitic sheets: The New Caledonia ophiolite (SW Pacific) as a case study? *Bulletin de la Société géologique de France* **184**, 545-556 (2013).
31 Searle, M. & Cox, J. Tectonic setting, origin, and obduction of the Oman ophiolite. *Geological Society of America Bulletin* **111**, 104-122 (1999).
32 Searle, M. P. Structural geometry, style and timing of deformation in the Hawasina Window, Al Jabal al Akhdar and Saih Hatat culminations, Oman Mountains. *GeoArabia* **12**, 99-130 (2007).
33 Schmid, S. M. *et al.* Tectonic units of the Alpine collision zone between Eastern Alps and Western Turkey. *Gondwana Research* (2019).
34 Whitehead, J., Reynolds, P. H. & Spray, J. G. The sub-ophiolitic metamorphic rocks of the Québec Appalachians. *Journal of Geodynamics* **19**, 325-350 (1995).
35 Schroetter, J. M., Bédard, J. H. & Tremblay, A. Structural evolution of the Thetford Mines Ophiolite Complex, Canada: implications for the southern Québec ophiolitic belt. *Tectonics* **24** (2005).
36 Schroetter, J.-M., Tremblay, A., Bédard, J. H. & Villeneuve, M. E. Syncollisional basin development in the Appalachian orogen—the Saint-Daniel Mélange, southern Québec, Canada. *GSA Bulletin* **118**, 109-125 (2006).
37 Chemenda, A., Matte, P. & Sokolov, V. A model of Palaeozoic obduction and exhumation of high-pressure/low-temperature rocks in the southern Urals. *Tectonophysics* **276**, 217-227 (1997).
38 Glodny, J., Bingen, B., Austrheim, H., Molina, J. F. & Rusin, A. Precise eclogitization ages deduced from Rb/Sr mineral systematics: the Maksyutov complex, Southern Urals, Russia. *Geochimica et Cosmochimica Acta* **66**, 1221-1235 (2002).
39 Pourteau, A. *et al.* Neotethys closure history of Anatolia: insights from 40Ar–39Ar geochronology and P–T estimation in high-pressure metasedimentary rocks. *Journal of Metamorphic Geology* **31**, 585-606 (2013).
